# Prickly Defenders: A Review of Venomous Sea Urchins (Echinoidea)

**DOI:** 10.3390/md23060253

**Published:** 2025-06-13

**Authors:** Sina Ehlert-Flaskämper, Cherie A. Motti, Richard J. Harris

**Affiliations:** 1 College of Science and Engineering, James Cook University, Townsville, QLD 4814, Australia; 2 AIMS@JCU, Division of Innovation, James Cook University, Townsville, QLD 4814, Australia; c.motti@aims.gov.au; 3 The Australian Institute of Marine Science (AIMS), Cape Cleveland, QLD 4810, Australia

**Keywords:** urchin, echinoidea, echinoderms, venom, toxin, defence

## Abstract

Sea urchins, Echinoidea, are widely known for their defensive spines and pedicellariae, with some species having co-evolved venom in conjunction with those appendages. Despite this, their venomous arsenal remains poorly understood. Research has predominately focused on pedicellariae venom, while the spines have been largely neglected within studies. This review consolidates current knowledge of the venom systems (spines and pedicellariae) of sea urchins, focusing on the morphology, known venom components, and their functional effects. While early studies have established the bioactivity of crude extracts and fractions, along with the partial characterisation of some toxins, most of these studies are outdated and were conducted with very basic methodologies. Modern venomics presents an opportunity to meet this challenge, enabling development of a comprehensive database on venomous urchins and their toxins. This advancement will facilitate research into targeted early treatments and therapies for victims of sea urchin stings, ultimately improving health outcomes and enhancing our scientific understanding of venom toxins and their broader implications for human health and bioinnovation.

## 1. Introduction

Echinoidea, commonly known as sea urchins, are a class of echinoderms whose common ancestor appeared in the fossil records in the Permian era and today includes around 1000 known extant species [1,2,3]. Their extensive distribution across all seas, from tropical to arctic regions, and their habitation of a wide range of environments, from shallow intertidal zones to bathyal and abyssal depths [4], underscore their significant role in benthic marine communities. As grazers of algae, sea urchins help maintain the balance of marine ecosystems by preventing algal overgrowth, which can otherwise dominate, disrupt, and change habitats.

**Figure 1:**
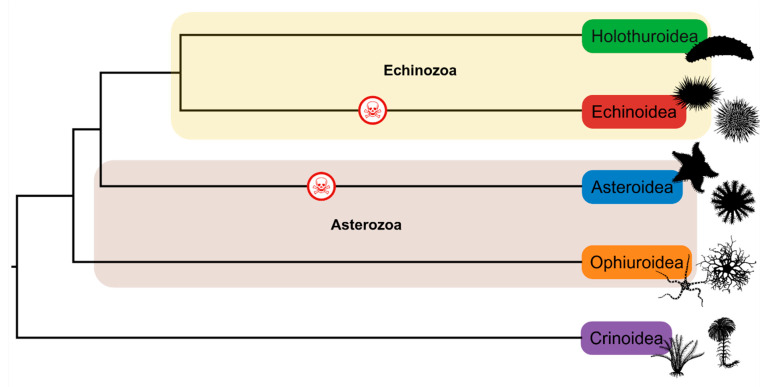
Phylogeny showing the five groups of echinoderms. The yellow highlighted lineages represent the Echinozoa clade, whilst the brown highlighted lineages represent the Asterozoa clade. The red skull indicates lineages that are known to have venomous members. Phylogeny was sourced from timetree.org, organism silhouettes were sourced from phylopic.com, and the figure was created using Biorender.com.

Echinoids are one of the five main clades that constitute the phylum Echinodermata (asteroids, crinoids, echinoids, holothuroids, and ophiuroids) [5,6]. They are a sister taxon to holothuroidians (sea cucumbers) within the clade Echinozoa (Figure 1). A defining primary morphological feature of Echinoidea (and some close echinoderm relatives) is the possession of a calcium carbonate (magnesium-rich calcite) skeleton that exhibits pentaradial symmetry (baring some exceptions, e.g., irregular urchins). Sea urchins are also relatively slow-moving organisms, relying on a water vascular system for movement and locomotion, making them highly vulnerable to predation. For many species, the most notably evolved defensive structures are spines and pedicellariae (small jaw-like appendages; see Section 3.2), and are the inspiration for the name Echinoidea, which is derived from the Greek word *echinos,* meaning ‘spiny’. These adaptations reduce predatory pressures from natural predators such as fish, otters, cetaceans, sea stars, and by proxy, humans, and in some urchin species, their evolution has enabled further evolutionary co-adaptation of innovations [7], the most notable being the creation of a venom system able to actively deliver a toxin through a sting. Note, here the definition of venom is as follows, “*a biological substance produced by an organism that contains molecules (“toxins”) which interfere with physiological or biochemical processes in another organism, which has evolved in the venomous organism to provide a benefit to itself once introduced to the other organism. The venom is produced and/or stored in a specialized structure and actively transferred to another organism through an injury by means of a specialized delivery system*” [8].

Only one genus of Asteroidea (*Acanthaster* species complex [9,10,11]) and some select Echinoidea have evolved a venom system, whereas Holothuroidea utilise toxins as poisons or noxious compounds, e.g., holothurinogenins and saponins [12,13,14,15,16]. *Acanthaster* sp. complex remains unique as they are known to produce toxins from venom apparatus and also secrete toxic saponins as poisons/deterrents [14,17].

Many authors have noted the neglect of marine venoms across different organisms [18,19,20,21], underlining the practical and conceptual challenges in their discovery and identification. These include collection and maintenance of marine organisms, complicated venom extraction techniques due to the small size or soft bodies of organisms, mucus contamination, and the instability of marine venoms under changing conditions such as heat or pH. Nevertheless, with increasing advancements in the fields of toxinology and venomics [22,23,24,25], new analytical tools are now available and, with this, a growing recognition of and a renewed effort in exploring marine venom systems for ecological and biotechnological application.

In contrast, relatively little is known about venoms across echinoderms, and in recent years, the exploration of their toxins has actually dwindled [21]. This is despite the fact that sea urchins, representing the group of echinoderms with the most venomous species, are responsible for some of the more common marine injuries to humans [26,27]. Further, it is even more concerning that venomous urchins have not been explored given that their close venomous asteroidea relative, *Acanthaster* species complex, are an ecologically devastating animal across coral reef systems [28]. As such, there remains fundamental gaps in knowledge regarding their venom apparatus, the toxins they produce, and the functional pathophysiology of these toxins.

This review provides a consolidated account of our current understanding of venom across Echinoidea, particularly focusing on the toxins that have been isolated, along with their biochemistry and functions. It also highlights the evolutionary and ecological importance of such toxins and provides a perspective on future research focussing on unravelling the toxic arsenal of Echinoidea and, by extension, echinoderms.

## 2. Venom System

There are two known venom producing apparatus within urchins: spines and pedicellariae [4,29]. Although both spines and pedicellariae play a mechanical defensive role—with or without the adoption of a venom—their form, function, and evolution are seemingly different.

### 2.1. Spines

Many sea urchin species have primary (longer) and secondary (shorter and morphologically and molecularly distinct) spine structures that occupy specific patterned areas of the test—the calcareous endoskeleton comprising interlocking plates [30]. The spines are attached to the tubercules of the test by muscle tissue, where they can be articulated via a ball-and-socket joint [30]. In addition to defence, they can also play a role in locomotion, posture, brooding, burrowing/boring, cleaning, and handling of objects on their test, e.g., a behavioural trait of Collector urchins [4].

**Figure 2:**
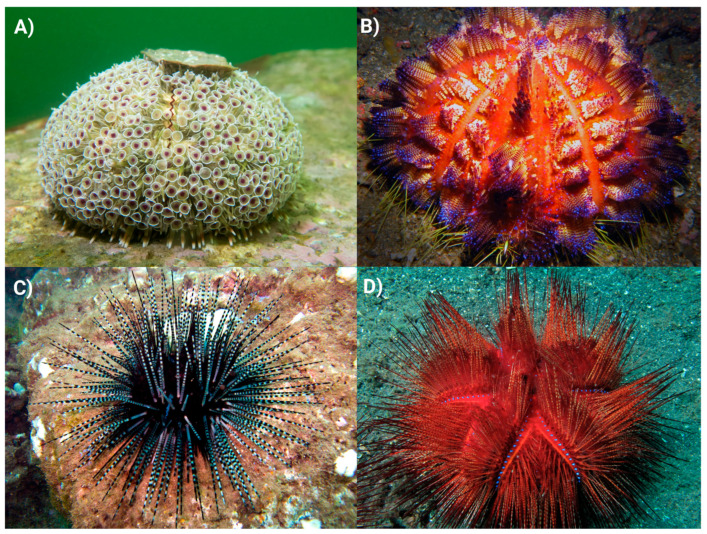
Images of four sea urchin species known to inflict medically painful stings. (**A**) *Toxopneustes pileolus*—©John Turnbull CC BY-SA 2.0; (**B**) *Asthenosoma varium*—©Pauline Walsh Jacobson CC BY-NC 2.0; (**C**) *Echinothrix calamaris*—©Ken-Ichi Ueda CC BY-SA 2.0; (**D**) *Astropyga radiata*—©Bernard Dupont CC BY-SA 2.0. All images were taken from flickr.com and reused in accordance with Creative Commons licences.

**Figure 3:**
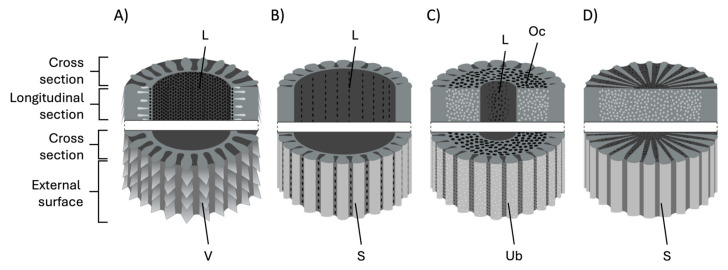
Generalised schematic cross-sectional diagram of defensive (aboral) spines reported across different urchin species. (**A**) spine with distinct verticillations (V) and a central lumen (L) capable of containing venom producing cells; (**B**) spine with a smooth external surface (S) and a large central lumen capable of containing venom producing cells; (**C**) spine with a small lumen surrounded by an outer cortex (Oc) with underdeveloped barbs (Ub) on the outer surface; (**D**) spine lacking a central lumen with a smooth external surface. Spine structures were adapted from [31,34,36,37,38,39,40] and created in Biorender.com.

Within echinoids, only diadematoids (e.g., genera such as *Diadema* spp., *Echinothrix* spp.) and echinothurioids (e.g., *Araeosoma* spp., *Asthenosoma* spp.) have been described as having members that possess venomous spines [31,32] (Figure 2). The spines are typically straight and taper at the distal tip to form a sharp needlepoint [31,33,34]. The cross section is circular, the lumen hollow and the stereom highly porous. In diadematoids, the hollow lumen and porous stereom are thought to harbour venom producing cells that then secrete the toxins to coat the outer surface of the spine or be distributed inside the wound [31,35] (Figure 3A). This has also been suggested for the closely related venomous asteroid species *Acanthaster planci* [11]. The true source of venom toxins within diadematoid spines remains to be fully elucidated, although one study has reported that the venomous tissue is likely the pigmented epidermal cells found both internally and externally on the spines of *Diadema* and *Echinothrix* urchins [33].

Spines can also differ in their surface ornamentation with some being relatively smooth and others highly serrated. These serrations allow for easy penetration of a target but make removal difficult, often breaking off within the wound [18] and causing further medical complications [35]. Along with serration, the brittle nature of these spines promotes breakage but might also, in part, be an evolutionary adaptation—favouring breakage in the wound to allow for maximal toxin distribution into the organism [35].

**Figure 4:**
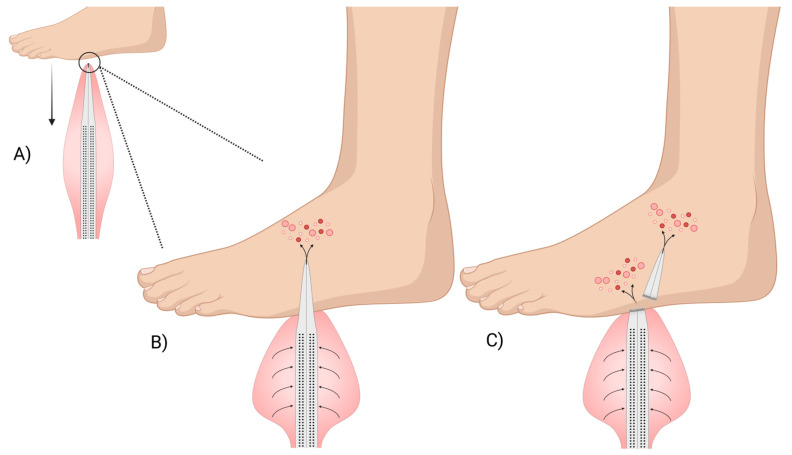
Diagram showing the hypothesised mechanism by which the venomous spines of *Araeosoma* and *Asthenosoma* urchins distribute venom. (**A**) The perforated spine is surrounded by an integumentary sheath filled with venom. (**B**) The needlepoint tip of the spine penetrates the foot, with the venom entering the hollow lumen via the pores and then travelling to the tip and into the wound. (**C**) The tip of the spine can break off within the wound, with the damaged spine still able to deliver venom into the victim. Figure has been adapted from Emson and Young (1985) [36]. Image was created using Biorender.com.

Conversely, the spines of Echinothuriidae members of the genera *Araeosoma* and *Asthenosoma* are covered with a fleshy integumentary sheath that is thought to contain the venom [34,36,41]. This spine structure represents a novel morphological adaptation not seen in other echinoid species. The venomous spines taper at the end like a hypodermic needle and the main shaft is perforated with pores that lead to a hollow lumen [34,36] (Figure 3B). Emson and Young (1998) [36] hypothesised that these pore structures and hollow lumen, in conjunction with the venom-filled integumentary sac, might aid in effective venom distribution into the victim. An adapted and modernised version of their hypothesis is illustrated in Figure 4. Again, little is known about this venom system and how or where the toxins are produced.

It is not yet known whether the evolution of the venomous arsenal in echinothurioids is related to their reduced skeletal development, i.e., less compact tests compared to other urchin species which equates to diminished structural robustness and necessitates an alternative form of defence [31,32]. Deep-sea echinothuroids, i.e., those that inhabit abyssal and bathyal zones, are uniquely representative of this trait, with many having a decalcified test that is maintained by positive water pressure, and which when removed from water results in a deflated sac-like appearance [42].

Furthermore, there remain significant gaps within the literature regarding any rigorous analysis of the types of cells or cellular tissues that contribute to venom production across urchin spine structures. This is an area in which, once uncovered, would not only provide valuable insights into the evolution of venomous spines, but would allow for a preliminary assessment of whether an urchin might be venomous based on their spine morphology, a notion that has also been suggested for venomous fishes [18].

### 2.2. Pedicellariae

Alongside the spines, echinoids have smaller stalked pincer-like appendages known as pedicellariae [29]. Some early observations of pedicellariae in sea urchins noted them as parasitic polyps (Müller, 1788 as noted in [43]). In later studies, they were in fact confirmed to be appendages [44], playing a defensive role, with some also featuring toxic secretions and containing venom glands [45]. Their evolution is independent of pedicellariae found in some asteroidea [46,47], suggesting they likely evolved from different spine structures [29]. See [43,48] for more details on the anatomy of pedicellariae.

**Figure 5:**
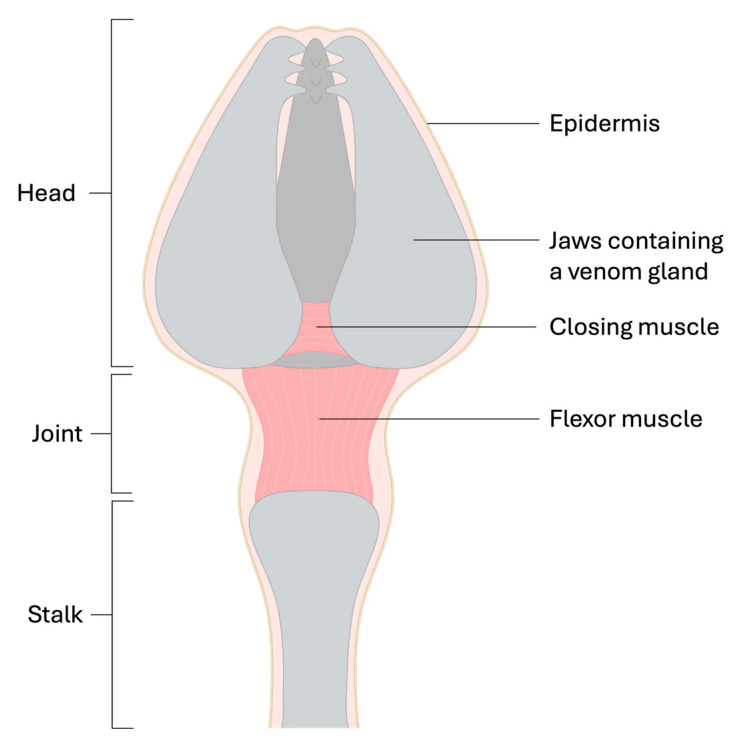
Schematic diagram of the globiferous pedicellariae showing position of the venom glands.

Echinoid pedicellariae can be categorised into four main structural types: globiferous (gemmiform or glandular), ophiocephalous, tridentate (tridactyle), and triphyllous (trifoliate) [29,48]. The main delineation between the type is based on morphology. All four types can often be observed on the same specimen, implying that they each might serve a different ecological purpose [29]. Coppard et al. (2012) [29] noted that the location and density on the test, the habitat in which the echinoid lives (e.g., epifaunal or infaunal), and the morphological characteristics of the different pedicellariae types, all provide clues as to their ecological role. It has been proposed that the ophicephalous types are likely for defence against smaller parasites and predators (e.g., nematodes, polychaetes); triphyllous types likely assist in freeing the epidermal surface of bacteria and small particulate matter; and tridentate have a dual a role in both cleansing and small parasite defence. The globiferous types are more likely for physical defence, providing protection against larger predators such as crustaceans, gastropods, octopus, fish, and even mammals (e.g., cetaceans). The globiferous type (Figure 5) are known to be venomous, containing distinct venom glands for toxin production [29]. Other pedicellariae subtypes of ophicephalous and tetradactylous (tridentate) have been found to contain glands in *Diadema* and *Araeosoma*, respectively [29,49]; however, there is little evidence that these glands functionally contribute to toxin delivery. They appear to be the exception to the rule within their type, as most other ophicephalous and tetradactylous do not seem to contain such glands.

As this review is specifically concerned with venom, only the globiferous pedicellariae (GP) will be discussed herein, them being (i) the more highly specialised of the four types regarding their venom apparatus, (ii) the most extensively researched regarding their evolved defensive capabilities, and (iii) seemingly containing more venomous subtypes than other pedicellariae groups. For an in-depth review of pedicellariae, see Coppard et al. (2012) [29].

The GPs are constructed of three jaws that are connected at their base to form a ‘head’ (Figure 5). The head is supported by a rigid stalk which allows for articulation via a ball-and-socket joint [29,48,50]. The jaws can move in tandem in a pincer-like fashion, and movements such as grabbing, pinching or ‘biting’ are controlled by muscles which are responsible for opening and closing the jaws. Each jaw has a sharp tooth extending from the epidermis at the distal portion and an associated venom gland structure. Jaw contraction, which is controlled by variously orientated criss-crossed muscle fibres, releases the venom from the venom-producing glandular epithelium which is then transported to a distally located duct that is embedded along a groove on the upper portion of the primary tooth [29,48,50].

The pincer-like action of the GP is dependent on chemical and tactile stimulation of cilia that form a sensory hillock [51,52]. The ejection of the venom is, on the other hand, independent of the closing of the jaws [43,53]. This separate distinct mechanism of venom ejection likely acts as a preventive measure against accidental venom loss when the jaws are stimulated by non-target particulates and organisms [29].

A unique adaptation has arisen in some species, such as the Collector urchin *Tripneustes gratilla*, whereby they can detach their GP and eject a cloud of venomous pedicellariae. Furthermore, these detached GP retain functionality, albeit with limited reflex control [43,48,53,54] providing a pre-emptive pursuit-deterrent mechanism [54]. It is thought that this adaptation might allow *T. gratilla* to successfully shift ecological niche to more diurnal foraging in the open [54]. Only one other urchin, *Lytechinus variegatus*, has been observed detaching their pedicellariae [55]. It remains unclear whether this defensive adaptation is unique to these two species or if other urchins that possess GP also possess this capability. On the other hand, species such as *Echinometra mathaei*, *Heliocidaris erythrogramma*, *Heliocidaris tuberculata*, and *Centrostephanus rodgersii*, failed to eject pedicellariae under experimental conditions [54]. While the extent to which different species can actively release GPs remains uncertain, regeneration of these structures has been documented, i.e., new GPs grew within 25 to 40 days after removal from *Psammechinus miliaris* [56].

The evolutionary history of GPs is largely unexplored. The emergence of primitive forms in the late Palaeozoic suggests an increase in predatory threats to echinoids. GPs underwent further evolutionary refinement in the early Mesozoic with the development of venom glands and likely represents an adaptive defence response to the Mesozoic marine revolution, characterised by the evolution of shell-crushing and boring predatory adaptations [29,57]. However, some closely related members of Asteroidea also have pedicellariae, e.g., *Acanthaster planci* and *Marthasterias glacialis* [46,47,58,59,60], but these appear to have originated independently [29]. Compared to echinoid pedicellariae, they are typically bivalved, smaller, non-stalked, and do not appear to possess any venom gland-like structures. Given that *A. planci* have venomous spines and produce other chemical defences, such as saponins [14,61,62], the evolution of venomous pedicellariae in Asteroidea might have been unnecessary.

## 3. Venom Toxins

### 3.1. Spine Venom

#### 3.1.1. *Echinothrix calamaris* and *E. diadema*

Toxins from the spines of the Diadematidae urchins *Echinothrix calamaris* and *E. diadema* were isolated and bioactivity established nearly 60 years ago [35]. Extracts of primary spines (i.e., long and sharp) were not bioactive, whereas those from secondary spines (i.e., short and sharp) were. When administered intravenously into anesthetised cats, the secondary spine extracts caused inconsistent increases and decreases in blood pressure across individuals with subsequent decrease in heart and respiration rate. The active substance was thermostable (up to 100 °C), acid-stable at low pH (pH 2), and dialysable. Noradrenaline was tentatively deduced as the bioactive candidate molecule within the secondary spine extract; serotonin was also hypothesised, based on the activity profile, but was not detected. Further investigation of the crude spine toxin extracts suggests that an unknown toxin(s) within the venom is responsible for triggering pain and bioactive effects. No attempts to further characterise the toxins from these species has been conducted since.

The absence of bioactive molecules in the primary spines suggests they function as a primary defensive barrier to inflict mechanical damage and give an early warning to predators [31]. Although shorter, the additional release of toxins by the secondary spines upon contact with the predator imparts an extra level of defence, thereby increasing the overall defensive output during an attack [35]. From an evolutionary perspective, having this multifaceted arsenal potentially limits the energetic demand on toxin maintenance and replenishment while still maintaining sufficient defensive capabilities [63]. It is, however, possible that the biochemical characterisation techniques available at the time meant the isolation and detection of any bioactive substances, especially those at very low concentrations, were simply missed. Recent advancements in biochemical analysis could illuminate previously undetected toxins in the primary spines, providing deeper insights into their chemical composition and potential bioactivity.

#### 3.1.2. *Echinometra mathaei*

The venom of *Echinometra mathaei*, a widely distributed sea urchin species in the Persian Gulf, has garnered some interest due to its potential pharmacological properties [64,65]. A biochemical analysis of spine-derived crude venom identified 12 distinct compounds via gas chromatography–mass spectrometry (GC-MS). These included biologically active molecules from a range of chemical classes, such as amides, alkaloids, terpenoids, alkanes, fatty acids, esters, and steroids, many of which are known for their neuroactive, cytotoxic, or anti-inflammatory effects.

Noteworthy among the detected compounds is harmine, a β-carboline alkaloid with well-documented acetylcholinesterase (AChE) inhibitory activity. Originally isolated from *Peganum harmala*, harmine has been shown to cross the blood–brain barrier and improve memory function in murine models of neurodegeneration [66]. It binds directly to the active site of AChE, thereby enhancing cholinergic neurotransmission, which may contribute to its therapeutic potential in conditions such as Alzheimer’s disease [66]. The presence of harmine in *E. mathaei* spine venom suggests that this species may possess neurotoxic capabilities aimed at predator deterrence, while simultaneously representing a promising source of bioactive marine natural products.

Moreover, functional assays were restricted to measuring the inhibition of cholinesterase enzymes (AChE and Butyrylcholinesterase (BChE)). While they are valuable pharmacological targets in the context of neurodegenerative disorders, these assays cannot measure other key venom functions, such as interactions with ion channels, nociceptive and inflammatory pathways, or cytolytic and immunomodulatory effects. Thus, while *E. mathaei* spines clearly harbour compounds of pharmacological interest, the full breadth of their biological activity and ecological function remains largely uncharacterised.

#### 3.1.3. *Echinometra lucunter*

*Echinometra lucunter* is the most abundant sea urchin species along the Brazilian coastline and is frequently involved in marine envenomation accidents, particularly in shallow reef and tide pool habitats [67]. Clinical reports consistently describe symptoms such as immediate and intense pain, inflammation, erythema, and oedema following spine punctures [27]. These effects, long thought to be solely mechanical in origin, are now understood to involve bioactive molecules embedded within the spines themselves.

An earlier study by Sciani et al. (2013) revealed that spine extracts of *E. lucunter* exhibit cysteine peptidase activity, specifically attributed to cathepsin B/X, as evidenced by substrate-specific cleavage of fluorogenic and FRET peptides, selective inhibition by E-64, and confirmation via Western blotting using anti-cathepsin B antibodies [60]. The term “cathepsin B/X” reflects the enzyme’s dual cleavage pattern as it cuts after both arginine and alanine residues, where cleavage after arginine is characteristic of cathepsin B, and cleavage after alanine is typical for cathepsin [68]. Although cathepsin B/X plays a role in tissue remodelling and inflammatory response [69], it remains uncertain as to whether it is also involved in sea urchin chemical defence.

Interestingly, a peptidomic profiling study applying MS and MS/MS based peptide de novo sequencing [70] was unable to detect any peptides in *E. lucunter* spine extracts. The authors attributed this to a combination of low peptide abundance, high hydrophilicity, and potential analytical limitations of the method, all of which may have resulted in a lack of retention of small or polar peptides during reverse-phase chromatography.

Another bioactivity-guided study of *E. lucunter* spine aqueous extracts, employing solid phase extractions (SPE) and direct injection MS and MS/MS, identified the main active constituent, nominally referred to as p3E [71]. This small molecule, with a mass of approx. 598 Da, induced robust hyperalgesia in rats and leukocyte adhesion and migration in mice [71]. Although the molecular identity of the compound remains uncertain, its proinflammatory and nociceptive effects were clearly demonstrated in vivo. Intravital microscopy revealed a significant increase in adherent and migrating leukocytes in the mouse cremaster muscle following subcutaneous injection of p3E, while paw pressure tests indicated a lowered nociceptive threshold for up to four hours after administration. Paw oedema was also observed, although to a lesser extent. The discovery of p3E underscores the importance of integrating bioactivity-guided approaches with molecular techniques, as a strict peptidomic strategy may miss relevant non-peptidic venom components. Thus, while *E. lucunter* spine venom clearly contains biologically active substances capable of inducing pain and inflammation, their chemical nature remains only partially characterised.

#### 3.1.4. *Lytechinus variegatus* and *Arbacia lixula*

Through de novo sequencing, Sciani et al. (2016) [70] identified multiple peptides in the spines of both *A. lixula* and *L. variegatus*, several of which exhibit structural similarity to known antimicrobial and antiviral peptides. However, it remains unclear if these peptides are components of the venom, having a clearly evolved toxic function, or if they serve another role in the cellular contents of the spines. Further research is needed to confirm their function.

### 3.2. Pedicellariae Toxins

#### 3.2.1. *Toxopneustes pileolus*

*Toxopneustes pileolus* are unique amongst sea urchins in that their main form of defence is their large and somewhat unique venomous GP (secondary in other species) that are reminiscent of petals on a flower, inspiring their colloquial name—the Flower urchin. They have minimal non-venomous spines on their aboral surface that are almost entirely disguised by the large GP (Figure 2A).

Case reports of stings describe severe pain, syncopy, respiratory distress, partial paralysis of the lips, tongue, eyelids, and extremities [72,73]. There are also reports of divers in Japanese waters who drowned after being stung by *T. pileolus* (noted by Endean (1961) [74], however, the original articles (cited as Fujiwara, 1935 and Mortensen, 1943) were inaccessible for this review.

Early research on *T. pileolus* pedicellariae crude venom discovered at least two large peptide/protein chromatography peaks, one comprising multiple isoforms or similar molecular weight toxins [75,76,77]. The crude venom elicited the contraction of smooth muscle, increased capillary permeability and inhibited cardiac contractions. The action upon smooth muscle was not affected by atropine, tetrodotoxin, hexamethonium, or methysergide, suggesting that acetylcholine receptors (AChR), voltage gated sodium channels (Na_V_), and serotonin (5-HT1) receptors were not the target. However, the toxin action was partially inhibited by tripelennamine—a H_1_-receptor antagonist—suggesting that the mode-of-action might induce histamine release [76,77]. This was further corroborated by subsequent studies [75,78] that revealed a single toxic fraction was responsible for causing histamine release. Histamine release is a function of some defensive venom toxins which leads to inflammatory pain. Further investigation of this toxic fraction showed that it also inhibits carbachol-enhanced secretion of catecholamines by supressing nAChR-mediated Na^+^ and Ca^2+^ influx in adrenal medullary cells [79].

The main toxin components of *T. pileolus* venom have since been identified as the sea urchin lectins (SUL-I, II, and III) [80,81,82,83], phospholipase A_2_-like proteins (PLA_2_) (Contractin A and UT841) [84,85,86], and the holoprotein toxin (Peditoxin) [87].

Lectins are a family of proteins/glycoproteins consisting of many variant compounds that recognise and bind reversibly to carbohydrate sites [88,89]. They are widely distributed across many animal venoms and function as defensive compounds, e.g., inducing the release of inflammatory mediators by triggering immunological responses through the disruption of cellular systems [90,91].

SUL-I is a 32 kDa immunomodulatory L-rhamnose-binding lectin (initially identified as a galactose-specific lectin as there is also some binding affinity to galactose moieties) capable of inducing mitogenic, chemotactic, phagocytic, and cytotoxic effects [80,81,82,83,92,93,94]. For example, SUL-I displays chemotactic activity toward human polymorphonuclear leukocytes [95] and induces maturation of human immature monocyte-derived dendritic cells in vitro [94], indicating SUL-I might be a good candidate in dendritic cell-based vaccines for cancer immunotherapy. An isoform, SUL-IA [96] with similar bioactivity has also been reported, however, further structural validation is required. Regardless, these bioactivities highlight the potential for SUL-1 as a bioprospecting tool in immunological and leukocyte research.

SUL-II has been reported as a 23 kDa D-galactose specific lectin [95,97]. Closer investigation of the partial amino acid sequences revealed there was no shared sequence homology to SUL-I [82,95], and based on a 45% and 40% sequence similarity to Contractin A and UT841, respectively [85,95], SUL-II was reassigned as a PLA_2_. SUL-III, a 170 kDa homohexameric protein (28 kDa subunits), was identified as a L-rhamnose-specific lectin [81] displaying similar bioactive properties to SUL-I, such as haemagglutinating activity toward rabbit erythrocytes and mitogenic stimulation of murine splenocytes. However, comparison of partial amino acid sequencing showed no homology to SUL-I, SUL-II, Contractin A, or UT841 [81].

Contractin A is a 14.9 kDa toxin isolated from *T. pileolus* spines and has been shown to cause contractile responses in multiple isolated smooth-muscle assays [84,85], yet the elucidation and characterisation of this toxin has not been straightforward. Initially, data suggested the toxin to be a phospholipase C (PLC) based on its activity being inhibited by the PLC inhibitor 2-nitro-4-carboxyphenyl-N,N-diphenylcarbarnate (NCDC) but not by the PLA_2_ inhibitor mepacrine [85]. However, cDNA cloning and expression revealed the N-terminal amino acid sequence shares homology with that of secreted PLA_2_s from other organisms and thus is likely a Ca^2+^-dependant PLA_2_. This is further supported by the isolation of a second spine-derived Contractin A-like toxin, named UT841 (18 kDa) [86], possessing an almost identical N-terminal sequence, but with three additional terminal amino acids. There remains debate as to whether UT841 is Contractin A, or whether it is an isoform.

Peditoxin was also isolated from the GP of *T. pileolus*. It is unusual in that it is a conjugated protein (or holoprotein) comprising the apoprotein pedin (a 10 kDa cytochrome b-like heme protein) and the prosthetic group pedoxin (a 204 Da heterocyclic lactone structure formed from pyridoxal and glycine: 3-hydroxy-2-methyl-5-methoxy-4-pyridineformyl-glycyliden ester) [87]. Peditoxin can cause anaphylaxis and death at low doses (LD_50_ 70 µg/kg) in mice and pedoxin sedation and anaesthetic-like effects, whereas pedin, was inactive. Only at extreme dosages (LD_50_ 200 mg/kg) did pedoxin cause similar effects to peditoxin, suggesting that in the holoprotein the two components (pedoxin and pedin) act synergistically to elicit the desired effects. Yet, peditoxin’s mode of action and biological function has never been determined, nor has its structure been fully characterised, which is surprising given its seemingly unique characteristics as a venom toxin.

Seasonal profiling of the GP crude venom revealed there was no obvious correlation between protein concentrations and bioactivity (i.e., the contractile potency determined based on the contraction of guinea-pig ileum longitudinal muscle), with protein concentrations lowest during the reproductive season (August–April) [98], and contractile potency having a biphasic activity profile (i.e., peaking October–December and again May–June). Energetic demands are high during reproduction (e.g., gonadal development), and it maybe that there is an energetic trade-off between reproduction vs. chemical defence resulting in a reallocation of resources away from the upkeep and maintenance of venom toxins, vis-à-vis, resulting in a reduction in protein concentration [99,100]. The increase in venom potency during the reproductive season may afford protection during brooding or egg guarding [100]. This is highly speculative and, given that urchins are broadcast spawners, it is unlikely that this scenario applies. It is more likely that other mechanisms, such as sexual or ontogentic, might be responsible for venom variation.

#### 3.2.2. *Toxopneustes roseus*

With the exception of pedoxin (the prosthetic group of peditoxin in *T. pileolus*) [87], most of the early research on venom components of urchin pedicellariae has focused on the peptide/protein type toxins, with small organic non-peptide molecules often overlooked. In the case of *Toxopneustes roseus*, a concerted effort was made to investigate the non-peptide component of the venom [101]. Applying GC-MS and LC-MS/MS techniques, six compounds were detected: benzoic acid; 2-aminoethanol (MEA); 2-(dimethylamine) ethanol (DMAE); 1-(4-bromophenyl)-1-phenylethanol (BPPE); 2-[1-(4-bromophenyl)-1-phenylethoxy]-N,N-dimethylethanamine (EMB); and 2-[1-(4-chlorphenyl)-1-phenylethoxy]-N,N-dimethylethanamine (CLX). EMB and CLX are likely the venom components as these are the predicted biosynthetic products of the other four precursors (refer to the hypothetical biosynthetic pathway in [101]). Based on pharmacophore modelling, both are chemically related to local anaesthetic, antiarrhythmic, antidepressant, and other H1-antihistamine molecules known to selectively bind to disrupt human voltage-gated sodium (Na_V_) channels, which suggests that their main role may also be similar. Additional computer-generated docking simulations revealed that both EMB and CLX are capable of binding to the Phe1748 on domain IV of human Na_V_1.7 receptors [101]. The blocking of human Na_V_1.7 induces pain insensitivity, and is reflective of the local inflammatory and analgesic effects noted for *T. roseus* envenomations [101]. However, the Phe1748 is also conserved in both Na_V_1.8 and Na_V_1.9, and thus it is possible that EMB and CLX can also target these receptors. Yet, further research is needed to confirm the broader Na_V_ receptor targeting of both these molecules.

Somewhat surprisingly, no further studies have been reported on the characterisation of the peptide and protein toxins from *T. roseus* venom, meaning there is still not a complete picture of the overall chemical arsenal nor how these compare to other venoms within closely related species such as *T. pileolus*. Likewise, the investigation of small molecule venom toxins in other urchin species remains a neglected aspect within current venomics workflows, although some authors are advocating for the adoption of new innovative approaches such as metabolomics to expedite the research [102].

#### 3.2.3. *Tripneustes gratilla*

The venomous potential of the Collector urchin *Tripneustes gratilla* has long been recognised. An early anecdote by Mortensen (1943) (as noted by Alender (1964) [103]) describes a stinging incident with *T. gratilla* pedicellariae, “That this tiny pedicellaria could have no harmful effect I had always thought beyond question. Now it happened that, on my opening a specimen rather roughly, a piece of the test covered with these pedicellariae hit my naked arm at the elbow joint. A very intense pain, much worse than that caused by nettles, was felt immediately… for several hours the pain continued, and a wound almost as from burning caused by these pedicellariae did not heal completely till after more than a month.”

The in vivo intravenous, intraperitoneal, and subcutaneous injection of the crude *T. gratilla* venom in albino mice revealed an LD_50_ 0.85 mg/kg [104]. Subsequent bioactivity-guided analyses of SPE fractions found the active fractions to be non-dialyzable, thermolabile and pH stable. Further characterisation revealed that the venom induces the release of histamine and other pharmacologically active agents from isolated tissues of guinea pigs and rats, including intestinal, cardiac, and pulmonary preparations [105]. The toxin’s effects were concentration-dependent and reduced by moderate heating, confirming the thermolabile nature of the active compounds. In pharmacological blockade assays, histamine antagonists such as pyribenzamine and bromolysergide (also known as BOL-148) partially inhibited the response, while atropine had no effect. These findings support the involvement of histamine and serotonin-like substances, but not acetylcholine, in the venom’s mode-of-action. A follow-up study showed that three fractions exhibited enzymatic activity, with one also being kininolytic [106]. However, the exact role of these enzymes and the relationship between kininolytic activity and venom toxin function remains unclear.

A subsequent bioactivity-guided study by Mebs (1984) [107] isolated two peaks through size exclusion chromatography, with one lethal at LD_50_ 0.75 mg/kg when administered intraperitoneally in mice. The discrepancies in the two studies may be attributed to variations in methods and/or the vast geographical differences in the specimens collected (Alender et al. (1965) [104] collected from Hawaii, US and Mebs (1984) [107] from Aqaba, Jordan), the latter likely given geographical variation in venoms is a well-known ecological constraint [108,109]. Further characterisation of this particular toxin has not occurred; thus, its biochemical nature remains unknown.

A second prominent component of the venom TGL-I (*Tripneustes gratilla* lectin 1) was later identified as a 23 kDa Ca^2+^-independent heparin-binding lectin [95,110]. Similarly, no further research has been conducted on this lectin, which might have potential as a pharmaceutical to compete with other heparin-binding proteins.

#### 3.2.4. *Lytechinus variegatus*

The earliest pharmacological investigation of *L. variegatus* GP was carried out by Mendes et al. (1963) [111], who reported the presence of a cholinergic, acetylcholine-like compound in GP homogenates. This dialyzable substance induced characteristic responses in a range of model systems, including the guinea pig ileum, amphibian heart, and the sea urchin’s own protractor muscle tissues, which are known to respond specifically to acetylcholine and related molecules. Its activity was potentiated by cholinesterase inhibitors and abolished by atropine, strongly suggesting that the active agent mimics acetylcholine or is structurally similar. The compound was partially heat-labile and inactivated by alkaline hydrolysis, which is typical of many small peptides or labile neurotransmitters. While its precise chemical identity was not determined, the results provide strong functional evidence for the presence of neuroactive venom components within the GP.

#### 3.2.5. *Lytechinus pictus*

Toxic components isolated from *Lytechinus pictus* GP using size exclusion chromatography have been shown to exert specific neurophysiological effects. For example, a high molecular weight protein extract impacted the neuromuscular transmission in the lined shore crab (*Pachygrapsus crassipes*) [112] by causing a significant and dose-dependent reduction in the amplitude of the excitatory junction potential recorded from single muscle fibres of the crab’s extensor leg muscles. Interestingly, the resting membrane potential of the muscle fibres exhibited only a modest depolarization, suggesting that the primary effect of the extract was not on postsynaptic membrane integrity or excitability. While this study confirms the presence of proteinaceous, neuroactive neuromuscular toxins in the GP of *L. pictus*, their molecular identity and the venom profile remains to be elucidated.

#### 3.2.6. Briefly Noted Toxins from Other Urchin Species

Alender et al. (1965) [104] provides a summary of early studies conducted on the toxic effects of GP homogenates from *Sphaerechinus grattularis*, *Echittus esculentus*, *Paracentrotus lividus*, and *Psammechirrus miliaris*. However, these studies have proven difficult to obtain through conventional literature search methods and ascertaining their validity has proven problematic.

A toxin from an unknown species of diadematid urchin was reported [113] and found to increase the frequency of Miniature Endplate Potentials (MEPPs) at the neuromuscular junctions, with the authors concluding that the toxin actuates the release acetylcholine (ACh). The toxin’s likely mechanism of action was suggested to be via enhancement of the cationic permeability of the synaptic membrane. Although these spine-derived urchin toxins were determined to be non-saponins and non-proteinaceous, no further efforts have been made to fully characterise them.

For a summary of all known urchin venom toxins refer to Table 1.

## 4. Envenomation and Human Interactions

Numerous species of sea urchin represent a valuable food source across many human societies [114,115], their gonads being highly nutritious and calorie-rich; however, their wild collection by divers, snorkellers, and fishers commonly leads to a variety of human-related injuries [26,27,73,116].

Although injuries caused by venomous urchin spines and pedicellariae are not of great medical concern (cf. venomous snakebites) they can cause severe local inflammatory responses, temporary local paralysis, intense pain, paresthesias, hypotension, cardiac arrhythmia, respiratory distress, secondary infections, and delayed presentations such as degenerative arthritis (i.e., sea urchin arthritis) [26,27,73,117,118,119,120]. Further complications might arise due to the spine breaking off within the wound, requiring minor surgical interventions [26,121,122], and further infection might occur from common marine microbial species such as *Exophiala jeanselmei* [123], *Mycobacterium marinum* [117,124], *M. chelonae* [118], and *Pasteurella* spp. [125].

To date, only one study has consolidated the best medical practices for treating sea urchin injuries [26]. The current lack of understanding regarding which sea urchin species are venomous and which are not has unfortunately hindered the development of more effective and targeted medical first-aid. As a result, generalised treatment protocols are often applied that may be less effective for venomous species. Developing a more robust database on venomous urchins and their toxins will facilitate research into targeted therapies for victims of stings even where the species cannot be fully identified but where medical symptoms along with species ecological data (e.g., water depth, habitat, geographical location, etc.) might provide enough information.

Furthermore, other than anecdotal sources, there is limited verifiable documentation of firsthand experiences of victims of venomous urchin stings. The many medical case reports of urchin envenomations rarely provide firsthand accounts from the victim’s perspectives; rather, they focus on the clinical presentation, treatment protocols, and outcomes of the envenomations. This lack of personal narratives from sting victims limits understanding of the full impact of these injuries and the effectiveness of current medical interventions. Some studies have begun to explore firsthand experiences of sting victims from other venomous species such as fishes [126,127] and snakes [128], particularly regarding their experience with the pain and how the sting affected their life afterward. These experience-focussed studies not only consider the symptoms and physiological responses to venom to determine the appropriate medical intervention and on-going care of the patient but can also provide much needed consilience—crucial for improving understanding of venom toxins and how their biochemical activity impacts the broader scope of human health.

## 5. Conclusions

Highlighted here is the novelty of Echinoidea (sea urchin) venoms, with an emphasis on the significant gaps in current knowledge regarding their composition, chemical structures, seasonality, and mechanisms of activity. Our findings underscore the potential of these toxins for bioprospecting and bioinnovation, and an argument is made for a comprehensive understanding of their biochemistry which would not only reveal their evolutionary origins but also pathophysiological functions.

Echinoidea venoms have been historically neglected, with much of the existing knowledge stemming from outdated decades-old research. However, despite the preliminary nature of current research, the advent of venomics [20,102,129]—a suite of advanced techniques including proteomics, metabolomics, transcriptomics, genomics, morphology, and functional bioassays—offers a promising opportunity to thoroughly and rapidly characterise these venoms. This holistic approach could propel sea urchin venom research to the forefront of various scientific fields, including evolutionary biology and ecology, and chemical ecology through to aquaculture, fisheries and fish science, and therapeutic applications. However, further research remains crucial, especially given the environmental threats to echinoderms, such as pollution and climate change. Advancing our understanding of these organisms is not only vital for potential bioinnovations but also for their conservation and preservation.

## 6. Materials and Methods

Relevant publications were initially identified using systematic searches within Web of Science and Google Scholar. Initial broad searches were conducted using various combinations of the keywords: ‘venom’, ‘toxin’, ‘spines’, ‘pedicellariae’, ‘urchin’, ’echinoidea’, ‘echinoderm’, ‘echinodermata’. More refined searches of specific known venomous taxa were also surveyed alongside these keywords, such as *Toxopneustes pileolus*, *Tripneustes gratilla*, etc. There was no limit to the publication date of articles, however, there were some notable articles (briefly mentioned in this manuscript) cited in other works that were inaccessible for this review, likely because of their vintage. Further, only peer-reviewed articles from legitimate publishing journals were assessed (although there is a brief mention of a postgraduate thesis within this review).

## Figures and Tables

**Table 1:** Known toxins isolated from the spine or pedicellariae of sea urchin species. † denotes there are multiple toxins reported in the reference. - denotes data is unsubstantiated or not available. * denotes tentative assignment by the author. “ denotes the same information as the above row. # denotes the toxin is likely an isoform of the previous report (row). Sp = Spines, Ped = Pedicellariae.

Species	Toxin	Structural Family	Mass	Tested Effects	Apparatus	References
*Echinometra lucunter*	Cathepsin B/X	Peptidase	60–80 kDa	Inflammation in mice	Sp	[69]
	p3E	Small molecule	598 Da	Nociceptive response in rats; inflammatory responses in cremaster muscle of mice	Sp	[71]
*Echinometra mathaei*	†	Proteins and small molecules	†	Cholinesterase inhibition; antioxidant activity	Sp	[64]
*Echinothrix calamaris and Echinothrix diadema*	-	-	-	Stimulation of smooth muscle; pain; hypotension	Sp	[35]
	Noradrenaline *	Catecholamine neurotransmitter	169.18 g/mol	-		
*Lytenchinus pictus*	-	-	-	Reduced excitatory synaptic potential.	Ped	[112]
*Lytenchinus variegatus*	-	Acetylcholine-like substance	-	Effects on cholinergic system	Ped	[111]
*Tripneustes gratilla*	TGL-I	Lectin	23 kDa	Ca^2+^-independent heparin-binding	Ped	[95,110]
	-	-	25 kDa	-	Ped	[107]
	-	-	-	Respiratory distress; hypotension; death in rabbits	Ped	[103]
*Toxopneustes pileolus*	Peditoxin (Pedin + Pedoxin)	Holoprotein (Apoprotein + prosthetic group)	10.2 kDa (10 kDa + 200 Da)	Anaphylaxis-like shock and death in mice	Ped	[87]
	SUL-I	Lectin	32 kDa	chemotactic properties for guinea-pig neutrophils; mitogenic activity on murine T-lymphocytes; D-galactose- and L-rhamnose-specific binding	Ped	[80,81,82,83,92,93,94]
	SUL-IA #	Lectin	32 kDa	“	Ped	[96]
	SUL-II	First identified as a lectin but likely a PLA_2_	23 kDa	D-galactose-specific binding	Ped	[95,97]
	SUL-III	Homohexameric lectin	170 kDa (28 kDa subunits)	L-rhamnose-binding lectin; agglutination of rabbit erythrocytes; mitogenic stimulation on murine splenocytes	Ped	[81]
	Contractin-A	PLA_2_	14.9 kDa	Mitogenic stimulation on murine splenocytes	Ped	[84,85]
	UT841	PLA_2_	18 kDa	-	Ped	[86]
*Toxopneustes roseus*	CLX (2-[1-(4-chlorphenyl)-1-phenylethoxy]-N,N-dimethylethanamine)	Small molecule	376.7 g/mol	Likely analgesic effects through Na_V_ channel blocking	Ped	[101]
	EMB (2-[1-(4-bromophenyl)-1-phenylethoxy]-N,N-dimethylethanamine)	Small molecule	348.3 g/mol	“		

## Data Availability

No new data was used to create this manuscript.
